# The correlation between red cell distribution width to albumin ratio and all-cause mortality in critically ill patients with rheumatic diseases: a population-based retrospective study

**DOI:** 10.3389/fmed.2023.1199861

**Published:** 2023-10-16

**Authors:** Lijuan Yin, Jie Min, Lei Zhong, Qikai Shen

**Affiliations:** ^1^Department of Rheumatology, Huzhou Central Hospital, Affiliated Central Hospital Huzhou University, Huzhou, Zhejiang, China; ^2^Department of Intensive Care Unit, Huzhou Central Hospital, Affiliated Central Hospital Huzhou University, Huzhou, Zhejiang, China; ^3^Affiliated Huzhou Hospital, Zhejiang University School of Medicine, Huzhou, China; ^4^The Fifth School of Clinical Medicine, Zhejiang Chinese Medical University, Huzhou, China

**Keywords:** red blood cell distribution width to albumin ratio, critically ill patients with rheumatic diseases, intensive care unit, all-cause mortality, MIMIC-IV database

## Abstract

**Background:**

Patients with rheumatic diseases have an increased likelihood of being admitted to the intensive care unit (ICU), highlighting the importance of promptly identifying high-risk individuals to enhance prognosis. This study aimed to assess the correlation of red blood cell distribution width to albumin ratio (RAR) with the 90-days and 360-days survival rates among critically ill rheumatic patients.

**Methods:**

Adult rheumatic patients admitted to the ICU from the Medical Information Mart for Intensive Care IV (MIMIC-IV) database were included. The participants were categorized into two groups, survivors (*n* = 436) and non-survivors (*n* = 192), based on their 90-days survival outcome. The population was further classified into tertiles using RAR values, with RAR < 4.63 (*n* = 208), 4.63–6.07 (*n* = 211), and > 6.07 (*n* = 209). Kaplan–Meier curves were utilized to evaluate the cumulative survival rates at 90-days and 360-days. The association between RAR and mortality was assessed using restricted cubic splines (RCS) and multivariate Cox regression analysis. Additional subgroup analyses and sensitivity analyses were conducted to further explore the findings. Receiver operating characteristic (ROC) curves were generated to evaluate the predictive performance of RAR.

**Results:**

This study involved 628 critically ill patients with rheumatic diseases, and they had an all-cause mortality of 30.57% at 90-days and 38.69% at 360-days. Kaplan–Meier analysis showed a gradual decrease in both 90-days and 360-days cumulative survival with increasing RAR (χ2 = 24.400, *p* < 0.001; χ2 = 35.360, *p* < 0.001). RCS revealed that RAR was linearly related to 90-days and 360-days all-cause mortality risk for critically ill patients with rheumatic diseases (χ2 = 4.360, *p* = 0.225; χ2 = 1.900, *p* = 0.594). Cox regression analysis indicated that elevated RAR (> 6.07) was significantly correlated with mortality. The ROC curves demonstrated that an optimal cut-off value of RAR for predicting 90-days mortality was determined to be 5.453, yielding a sensitivity of 61.5% and specificity of 60.3%.

**Conclusion:**

Elevated RAR (> 6.07) was associated with all-cause mortality at 90-days and 360-days among critically ill patients with rheumatic diseases, serving as an independent risk factor for unfavorable prognosis.

## Introduction

As a chronic inflammatory disorder involving multiple systems and organs, rheumatic disease is usually accompanied by immunological disturbances. In addition, immunosuppressive medications are often required for treatment, so such patients are prone to severe acute multiple organ failure. Patients with rheumatic diseases have a higher risk and mortality rate of admission to the intensive care unit (ICU) compared to the general population (1, 2). The mortality rate of ICU population with rheumatic diseases has been reported to be 30.5–48% (3–5), which was greater than that of the common ICU patients (1, 6). Thus, early identification for rheumatic patients at high risk of mortality is critical to improving prognosis and optimizing healthcare resource utilization.

Red blood cell distribution width (RDW), that represents erythrocyte volume heterogeneity, increases during systemic inflammation and is useful for monitoring the disease activity in autoimmune diseases (7). RDW has been demonstrated to be a powerful indicator of all-cause mortality in critically ill patients (8), and it is also related to disease activity and outcomes in multiple rheumatic diseases (9, 10). An unclear mechanism, related perhaps to inflammation response, may underlie this process (11). Like C-reactive protein (CRP), RDW may be a useful tool when it comes to distinguishing from articular inflammatory and non-inflammatory joint diseases (9). Albumin, produced by the liver, is also known as the negative acute phase reactant and represents nutritional levels as well as inflammatory status (12, 13). Lower serum albumin levels are linked to clinical prognosis among critical illness, as per recent data (14, 15).

The ratio of red blood cell distribution width to albumin (RAR) is a simple and innovate biomarker of inflammation calculated from RDW and albumin. Previous research have demonstrated that RAR can be used as an important prognostic indicator among patients with sepsis (16), cancer (17), burn surgery (18), diabetic foot ulcers (19), heart failure (20), and acute respiratory distress syndrome (21). Rheumatic diseases are primarily distinguished by persistent inflammation and immune dysregulation (22), prompting us to hypothesize a potential association between RAR and the prognosis of this population. However, the absence of pertinent studies necessitates our own investigation into the correlation between RAR and all-cause mortality among ICU patients with rheumatic diseases.

## Materials and methods

### Data sources

Medical Information Mart for Intensive Care IV (MIMIC-IV, v2.0) database, which contains a substantial amount of free medical data, was used for this retrospective study. As a new version of MIMIC-III, MIMIC-IV contains 76,943 ICU admissions from 2008 to 2019 (23). We had permission to access this database (certification number: 51774135; 36142713). The Massachusetts Institute of Technology and Beth Israel Deaconess Medical Center provided their approval for the database. All personal identifiers have already been deleted to afford privacy protection for participants. Consequently, this study waived consent requirements for individual patients. We extracted eligible rheumatic patients from MIMIC-IV database and conducted the study based on the Strengthening the Reporting of Observational Studies in Epidemiology (STROBE) Statement (24).

### Study participants

Adult ICU participants (age ≥ 18 years) suffering from rheumatic diseases in the MIMIC database were enrolled in our study. We just adopted the initial one when the participants had multiple ICU admission data. The following were the exclusion criteria: (1) Died within one day after admission to ICU and (2) Missing critical information such as RDW and albumin.

### Variable extraction

To identify rheumatic diseases, we utilized the Charlson Index as outlined on the official website of the MIMIC database. Subsequently, specific rheumatic disease types were extracted from patient records using Structured Query Language (SQL) queries incorporating ICD-9 and ICD-10 codes. Variables were gathered as age, sex, Acute Physiology and Chronic Health Evaluation (APACHE) II score, sequential organ failure assessment (SOFA) score, coexisting diseases, additional acute organ dysfunction, continuous renal replacement therapy (CRRT) use, mechanical ventilation (MV) use, all-cause mortality at 90-days and 360-days, and lengths of stay (ICU, hospital). At the same time, laboratory parameters including RDW, albumin, red blood cell (RBC), white blood cell (WBC), platelet, mean corpuscular volume (MCV), anion gap (AG), serum creatinine (Scr), blood urea nitrogen (BUN), blood glucose, serum potassium, total serum calcium, serum phosphorus were extracted. The RAR was determined as follows: RDW (%) divided by albumin (g/dL). The SOFA score and laboratory variables were extracted based on data available during the initial 24 h after ICU admission.

### Groups and outcomes

The participants were categorized into two groups, survivors (*n* = 436) and non-survivors (*n* = 192), based on their 90-days survival outcome. Additionally, the population was further classified into tertiles using RAR values, with tertile 1 having RAR values less than 4.63 (*n* = 208), tertile 2 having RAR values between 4.63 and 6.07 (*n* = 211), and tertile 3 having RAR values greater than 6.07 (*n* = 209). The endpoints were all-cause mortality at 90-days and 360-days following admission to ICU.

### Statistical analysis

Continuous variables with normal distribution were presented as mean ± standard deviation (SD), while non-normally distributed variables were expressed as median (interquartile range, IQR). One sample *t*-test and Wilcoxon rank-sum test were applied for the analysis. Chi-square test was applied to examine categorical variables, which were described as numbers (%).

Kaplan–Meier curves were utilized to evaluate the cumulative survival rates at 90-days and 360-days for each group. The correlation between RAR and all-cause mortality risk was assessed using restricted cubic spine (RCS). Then we constructed multivariate Cox regression models that comprised covariates with *p* < 0.10 in the univariate analysis and Hazard ratios (HR) with 95% confidence intervals (CI) were expressed. We did not adjust Model I for any covariates. Model II was adjusted for malignancy, congestive heart failure (CHF), chronic kidney disease (CKD), acute kidney injury (AKI), sepsis, MV use, and CRRT use. Model III was adjusted for model II plus age, SOFA score, WBC, Scr, BUN, AG, potassium, and phosphorus.

Subgroup analyses were performed to determine the association between RAR and 90-days mortality across different subgroups, such as age, sex, malignant tumor, CKD, CHF, AKI, sepsis, and the use of MV and CRRT. Several sensitivity analyses were conducted to evaluate the reliability of our findings. Receiver operating characteristic (ROC) curves were also generated to evaluate the predictive performance of RAR.

We used Stata14.0 software along with R language (version 4.2.0) for analysis. The differences were regarded as statistically significant at a two-sided *p*-value less than 0.05.

## Results

### Study population and baseline characteristics

**Figure 1:**
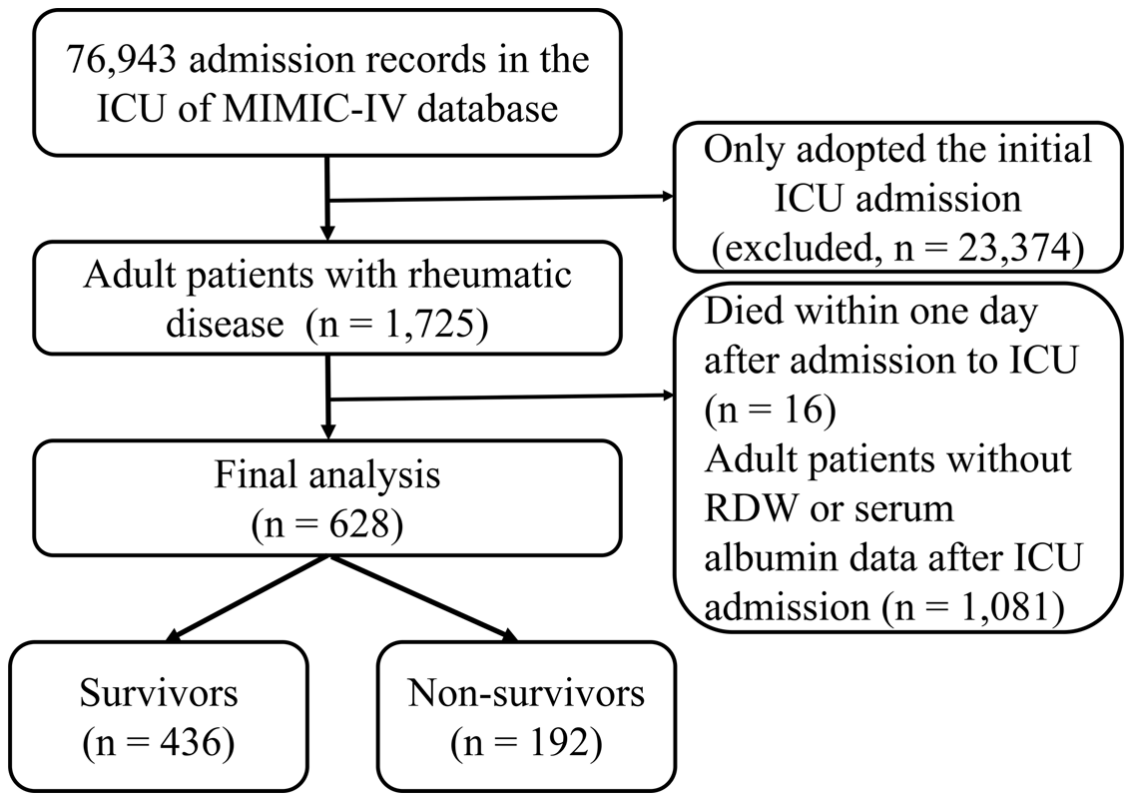
The flowchart for study participants enrollment.

The flowchart for study participants enrollment was noted in Figure 1. Ultimately, this study involved 628 critically ill patients with rheumatic diseases. There were 330 (52.55%) patients with RA, 122 (19.43%) patients with SLE, and 187 (29.78%) patients with other rheumatic diseases. The average age was 69.49 years among patients, and 70.20% of them were female. RAR had an average baseline value of 5.68 ± 1.94. The main acute organ dysfunction were sepsis (64.97%) and AKI (62.74%). Non-survivors had higher age, APACHE II score, SOFA score, RDW, RAR, WBC, Scr, BUN, AG, serum potassium, and serum phosphorus, compared with survivors. Among non-survivors, the incidence rates of malignancy, CKD, CHF, AKI, and sepsis were greater. In contrast, survivors had significantly higher albumin level and shorter ICU stay. The clinical characteristics and laboratory variables for study participants are listed in Table 1.

**Table 1:** The basic clinical characteristics and laboratory variables for study participants.

Variable	Overall population (*n* = 628)	Survivors (*n* = 436)	Non-survivors (*n* = 192)	t/Z/χ2	*p*
Age (years)	69.49 ± 15.82	67.09 ± 15.74	74.96 ± 14.62	−5.896	<0.001
Female, *n* (%)	441 (70.20)	312 (71.56)	129 (67.19)	1.219	0.270
SOFA score	5.00 (3.00, 8.00)	4.00 (2.00, 7.00)	7.00 (5.00, 11.00)	−7.675	<0.001
APACHE II score	22.26 ± 7.74	20.54 ± 6.83	26.19 ± 8.25	−8.946	<0.001
RDW (%)	15.80 ± 2.52	15.46 ± 2.30	16.57 ± 2.83	−5.175	<0.001
Albumin (g/dL)	2.96 ± 0.64	3.03 ± 0.63	2.79 ± 0.63	4.543	<0.001
RAR (%/g/dL)	5.68 ± 1.94	5.38 ± 1.63	6.37 ± 2.37	−6.128	<0.001
WBC (×10^9^/L)	11.00 (7.10, 15.70)	10.45 (6.80, 14.90)	12.20 (8.15, 17.55)	−2.829	0.005
RBC (×10^12^/L)	3.43 ± 0.70	3.45 ± 0.69	3.38 ± 0.72	1.187	0.236
Platelet (×10^9^/L)	194 (125, 266)	195 (127, 263)	188 (119, 275)	0.144	0.886
MCV (fl)	91.95 ± 7.06	91.86 ± 6.77	92.16 ± 7.70	−0.480	0.631
BUN (mg/dL)	22(14, 36)	20 (13, 32)	29 (19, 50)	−5.527	<0.001
Scr (umol/L)	88.40 (61.88, 150.28)	79.56 (61.88, 123.76)	106.08 (70.72, 176.80)	−3.987	<0.001
AG (mmol/L)	15.38 ± 4.24	14.96 ± 4.11	16.33 ± 4.38	−3.784	<0.001
Glucose (mmol/L)	6.97 (5.50, 9.08)	6.92 (5.50, 8.92)	7.08 (5.53, 9.31)	−0.788	0.430
Potassium (mmol/L)	4.24 ± 0.83	4.19 ± 0.84	4.33 ± 0.79	−1.965	0.049
Total calcium (mmol/L)	2.05 ± 0.23	2.06 ± 0.22	2.04 ± 0.24	0.676	0.499
Phosphorus (mmol/L)	1.26 ± 0.51	1.21 ± 0.47	1.37 ± 0.59	−3.500	<0.001
CRRT use, *n* (%)	41 (6.53)	18 (4.13)	23 (11.98)	13.463	<0.001
MV use, *n* (%)	355 (56.53)	215 (49.31)	140 (72.92)	30.224	<0.001
Type, *n* (%)					
RA	330 (52.55)	243 (55.73)	87 (45.31)	5.806	0.016
SLE	122 (19.43)	89 (20.41)	33 (17.19)	0.886	0.347
Others	187 (29.78)	114 (26.15)	73 (38.02)	8.988	0.003
Coexisting diseases, *n* (%)					
Hypertension	262 (41.72)	184 (42.20)	78 (40.63)	0.136	0.712
Diabetes	160 (25.48)	116 (26.61)	44 (22.92)	0.955	0.328
malignant tumor	69 (10.99)	33 (7.57)	36 (18.75)	17.040	<0.001
CKD	135 (21.50)	81 (18.58)	54 (28.13)	7.200	0.007
Chronic pulmonary disease	210 (33.44)	143 (32.80)	67 (34.90)	0.264	0.608
Myocardial infarct	126 (20.06)	81 (18.58)	45 (23.44)	1.963	0.161
CHF	215 (34.24)	133 (30.50)	82 (42.71)	8.818	0.003
Additional acute organ dysfunction, *n* (%)					
AKI	394 (62.74)	253 (58.03)	141 (73.44)	13.541	<0.001
Sepsis	408 (64.97)	255 (58.49)	153 (79.69)	26.326	<0.001
Length of ICU stay (days)	3.12 (1.72, 6.25)	2.86 (1.61, 5.51)	4.94 (2.02, 8.37)	−4.439	<0.001
Length of hospital stay (days)	9.42 (5.81, 16.75)	9.44 (6.29, 16.98)	9.31 (5.10, 15.33)	1.468	0.142

SOFA, sequential organ failure assessment; APACHE II, acute physiology and chronic health evaluation II; RDW, red blood cell distribution width; RAR, red blood cell distribution width to albumin ratio; WBC, white blood cell; RBC, red blood cell; MCV, mean corpuscular volume; Scr, serum creatinine; BUN, blood urea nitrogen; AG, anion gap; CRRT, continuous renal replacement therapy; MV, mechanical ventilation; RA, rheumatoid arthritis; SLE, systemic lupus erythematosus; CKD, chronic kidney disease; CHF, congestive heart failure; AKI, acute kidney injury.

### RAR and all-cause mortality

The all-cause mortality rates for the entire study population at 90-days and 360-days were 30.57 and 38.69%, respectively, as specified in Table 2. Using RAR values, the population was divided into tertiles (< 4.63, 4.63–6.07, > 6.07). Differences in 90-days mortality for the three groups were statistically significant (χ2 = 24.706, *p* < 0.001), and patients who had elevated RAR (> 6.07) died at a rate that was noticeably higher (42.11%). Similar results were obtained in the comparison of 360-days mortality among the three groups.

**Table 2:** Comparison of all-cause mortality among the three groups.

Variable	Total (*n* = 628)	RAR < 4.63 (*n* = 208)	RAR 4.63–6.07 (*n* = 211)	RAR > 6.07 (*n* = 209)	χ2	*p*
90-days mortality, *n* (%)	192 (30.57)	41 (19.71)	63 (29.86)	88 (42.11)	24.706	<0.001
360-days mortality, *n* (%)	243 (38.69)	50 (24.04)	83 (39.34)	110 (52.63)	35.985	<0.001

RAR, red blood cell distribution width to albumin ratio.

### Kaplan–Meier survival curve analysis

**Figure 2:**
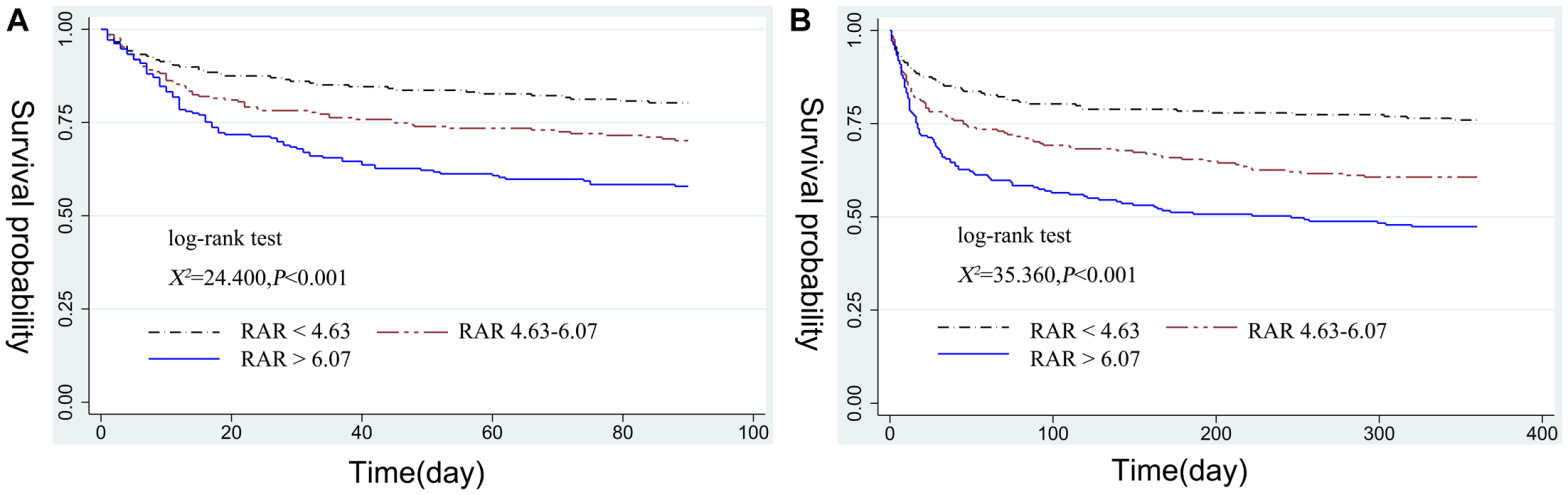
Kaplan–Meier curve of 90-days cumulative survival rates at various RAR values **(A)**. Kaplan–Meier curve of 360-days cumulative survival rates at various RAR values **(B)**. RAR, red blood cell distribution width to albumin ratio.

Stratified by RAR tertiles, we plotted 90-days and 360-days survival curves for patients to evaluate cumulative survival at various RAR values. As displayed in Figures 2A,B, Kaplan–Meier analysis showed a gradual decrease in both 90-days and 360-days cumulative survival with increasing RAR (log-rank test, χ2 = 24.400, *p* < 0.001; χ2 = 35.360, *p* < 0.001).

### Elevated RAR was significantly correlated with all-cause mortality

**Figure 3:**
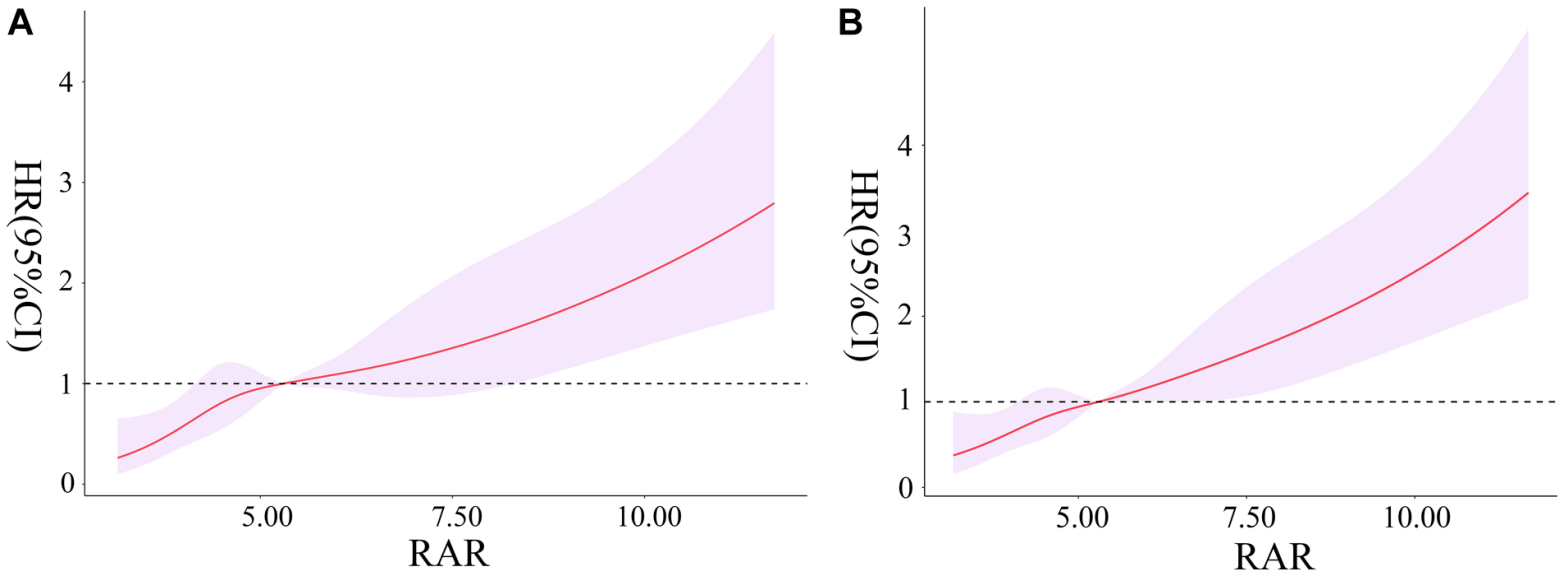
Association between RAR and the risk of 90-days all-cause mortality **(A)**. Association between RAR and the risk of 360-days all-cause mortality **(B)**. RAR, red blood cell distribution width to albumin ratio.

For critically ill patients with rheumatic diseases, it was observed that RAR was linearly related to 90-days and 360-days all-cause mortality risk (χ2 = 4.360, *p* = 0.225; χ2 = 1.900, *p* = 0.594). We observed a gradual increase in 90-days and 360-days mortality risk with increasing RAR, as indicated in Figures 3A,B.

In model I adjusted for not any variable, the HR (95% CI) of 90-days mortality for the second and third group were 1.610 (1.087–2.386) and 2.445 (1.687–3.543), respectively. In model III, even after adjustment for a range of confounders, the HR (95% CI) of 90-days and 360-days mortality were 2.016 (1.360–2.988) and 2.351 (1.649–3.352) for the third group. The multivariate Cox regression analysis indicated that elevated RAR (> 6.07) was significantly correlated with 90-days and 360-days all-cause mortality, as shown in Table 3.

**Table 3:** Cox regression analysis of the association between RAR and all-cause mortality.

Variable	Model I			Model II			Model III		
	HR	95%CI	*p*	HR	95%CI	*p*	HR	95%CI	*p*
90-days mortality									
RAR <4.63	baseline			baseline			baseline		
RAR 4.63–6.07	1.610	1.087–2.386	0.018	1.428	0.960–2.122	0.078	1.297	0.866–1.941	0.207
RAR > 6.07	2.445	1.687–3.543	<0.001	1.863	1.278–2.718	0.001	**2.016**	**1.360–2.988**	**<0.001**
360-days mortality									
RAR <4.63	baseline			baseline			baseline		
RAR 4.63–6.07	1.789	1.259–2.541	0.001	1.586	1.114–2.260	0.011	1.511	1.054–2.164	0.025
RAR > 6.07	2.656	1.901–3.713	<0.001	2.102	1.495–2.955	<0.001	**2.351**	1.649–3.352	**<0.001**

Model I was adjusted for no variables.

Model II was adjusted for malignancy, CKD, CHF, AKI, sepsis, MV use, and CRRT use.

Model III was adjusted for model II plus age, SOFA score, WBC, Scr, BUN, AG, potassium, and phosphorus.

HR, hazard ratio; CI, confidence interval; RAR, red blood cell distribution width to albumin ratio; CKD, chronic kidney disease; CHF, congestive heart failure; AKI, acute kidney injury; MV, mechanical ventilation; CRRT, continuous renal replacement therapy; SOFA, sequential organ failure assessment; WBC, white blood cell; Scr, serum creatinine; BUN, blood urea nitrogen; AG, anion gap.

### Subgroup analyses

**Figure 4:**
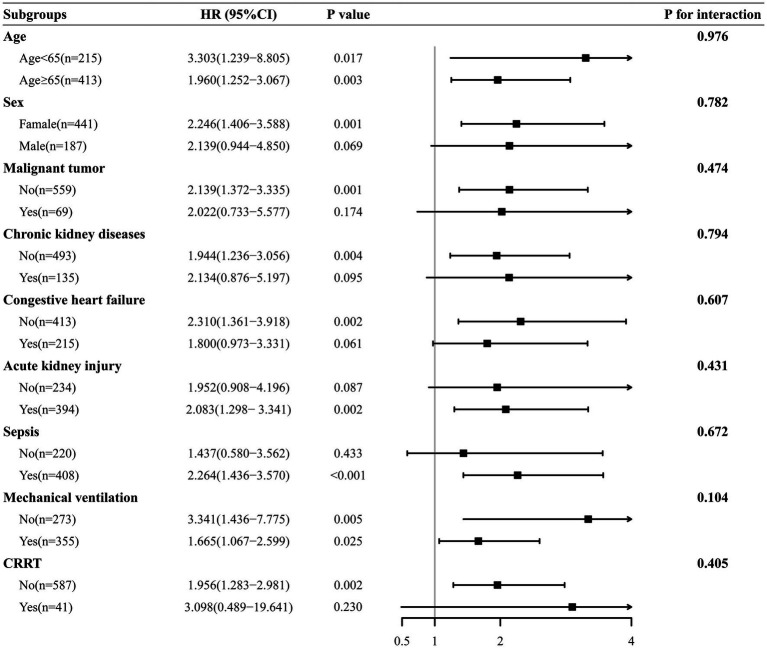
The association between RAR and all-cause mortality in the subgroups. RAR, red blood cell distribution width to albumin ratio.

Subgroup analyses were performed to determine the association between RAR and 90-days mortality across different subgroups, as shown in Figure 4. Multivariable Cox regression analysis in subgroups adjusted the same covariates as for Model III in Table 3. The results showed that in most sub-populations, increased RAR level was associated with 90-days all-cause mortality in critically ill rheumatic patients. Rheumatism has a clear predilection for women, and over 70% of included patients in our study were female. The result of subgroup analysis suggested that our finding was also applicable to the main population of patients (HR = 2.246, 95% CI: 1.406–3.588). Additionally, no significant interactions were observed in the subgroups (*p* for interaction > 0.05).

### Sensitivity analyses

Several sensitivity analyses were conducted to evaluate the reliability of our findings. Initially, we conducted separate COX regression analyses on 330 critically ill patients with RA, which indicated a significant association between elevated RAR (> 6.07) and all-cause mortality at both 90 and 360-days (Supplementary Table S1). Subsequently, a COX regression analysis was performed on 122 critically ill patients with SLE, but no significant findings were observed (Supplementary Table S2). Furthermore, the potential impact of human serum albumin infusion on the RAR value should be considered prior to admission to the ICU. To investigate this, a sensitivity analysis was conducted, excluding patients who had received human serum albumin infusion within 48 h before being admitted to the ICU. The findings revealed a persistent association between elevated RAR and unfavorable clinical outcomes in critically ill patients with rheumatic diseases (Supplementary Table S3). Additionally, to address the potential influence of reduced liver synthetic capacity, another sensitivity analysis was performed, excluding patients with cirrhosis. The results remained consistent with the initial finding (Supplementary Table S4).

### Comparative analysis of ROC curves

The ROC curves demonstrated that an optimal cut-off value of RAR for predicting 90-days mortality was determined to be 5.453, yielding a sensitivity of 61.5% and specificity of 60.3%. The area under the curve (AUC) was calculated to be 0.643. Notably, the predictive performance of RAR was very close to the APACHE II score and the SOFA score (RAR AUC = 0.643; APACHE II score AUC = 0.699; SOFA score AUC = 0.691). Furthermore, when RAR was combined with SOFA and APACHE II score, the predictive performance reached its peak (AUC = 0.733), accompanied by a sensitivity of 77.1% and specificity of 58.5% (Supplementary Tables S5, S6; Supplementary Figure S1).

## Discussion

Based on MIMIC-IV database, our current study explored the correlation between RAR and all-cause mortality for critically ill patients with rheumatic diseases. In contrast to survivors, we discovered that RAR was higher in non-survivors. Kaplan–Meier analysis revealed that 90-days and 360-days cumulative survival rates gradually declined with increasing RAR. It was observed that RAR was linearly related to 90-days and 360-days mortality risk. Further analysis of Cox regression indicated that elevated RAR (> 6.07) was significantly correlated with short- and long-term mortality among critically ill patients with rheumatic diseases. To our knowledge, this is the first study on the correlation between RAR, a novel integrative biomarker, and prognosis in critically ill patients with rheumatic diseases.

The mechanisms underlying the association between elevated RAR and rheumatic disease patients admitted in ICU have not been fully elucidated. Oxidative stress in the body and systemic inflammatory response can lead to the inhibition of erythropoiesis, the promotion of erythrocyte apoptosis, the reduction of iron metabolism, the modifying of erythrocyte membranes and the altering of erythrocyte morphology (25–30). These pathological changes result in increased heterogeneity of red blood cell volume, which manifests as an elevated RDW. Although RDW has historically been used to differentiate between anemic diseases, it has also been identified in recent years as an inflammatory marker among various disorders, such as sepsis (31), rheumatoid arthritis (RA) (32), autoimmune liver diseases (33), and systemic lupus erythematosus (SLE) (34). As the most prevalent protein in plasma, serum albumin is essential for preserving the colloid osmotic pressure of plasma (35). Rheumatic diseases may involve multiple systems, including the liver, which can be damaged and result in a reduction in albumin synthesis (36, 37).It is worth noting that albumin levels decrease as the infection progresses and are linked to inflammatory response and organ failure (15). Previous research has demonstrated a significant reduction in albumin-binding function among critically ill patients suffering from sepsis or septic shock (38).

The ratio of RDW to albumin, known as RAR, provides a comprehensive representation of the acute inflammatory response in patients. When compared to individual markers such as RDW and albumin, RAR offers a more composite assessment of disease progression. It was revealed in a study involving 14,639 sepsis participants that RAR was positively related to mortality at in-hospital, 28-days, and 90-days, as well as their ICU stay and hospital stay (16). Additionally, RAR has been demonstrated to be a powerful indicator of sepsis for patients with diabetic ketoacidosis (HR: 2.9, 95% CI: 2.0–4.1, *p* < 0.001) (39). However, no research has examined the correlation of RAR with prognosis for critically ill rheumatic patients. Our current findings showed that RAR and mortality at 90-days and 360-days were in correlation, with higher RAR predicting a worse clinical outcome.

Presently, the SOFA score, APACHE score, and SAPS score are widely employed in the ICU to evaluate patient prognosis. However, the APACHE II score encompasses numerous indicators and is comparatively intricate. Consequently, numerous scholars have endeavored to identify cost-effective and readily available prognostic markers of disease to enhance clinical practice. The present study contributes to the existing body of literature by confirming the association between elevated RAR, a novel comprehensive inflammatory index, and unfavorable prognosis in critically ill patients with rheumatic diseases. A study conducted on patients with critical pneumonia receiving MV support demonstrated that individuals with a RAR cutoff value > 5.73 exhibited a significantly higher 28-days mortality rate compared to those with a RAR cutoff value <5.73 (AUC 0.688). Similarly, another study focusing on patients with coronavirus diseases 2019 (COVID-19) revealed that the optimal cutoff value of RAR for predicting mortality was 5.43. Consistent with these findings, the ROC curve analysis in the present study indicated that the optimal cutoff value of RAR for predicting 90-days mortality was 5.453 (AUC 0.643). Furthermore, a comparative analysis was conducted on the ROC curves of the RAR, SOFA score, and APACHEII score. The findings revealed that the RAR exhibited a similar level of prognostic predictive ability as the latter two scores. Additionally, the combination of the RAR with the two scores enhanced their prognostic predictive power.

Our research showed that the most common acute organ dysfunction for ICU rheumatic patients was sepsis (64.97%), and the incidence of sepsis was higher (79.69%) for non-survivors. These findings suggested a relationship between infection and ICU admission in patients with rheumatic diseases. A multicenter retrospective study involving ten French ICUs also confirmed similar results. The leading cause for patients with rheumatic diseases to enter ICU was sepsis, with an incidence rate of 61.6% (40). For patients with rheumatic diseases, the mortality rate was higher in hospitalized patients with infectious causes than in those with non-infectious causes (3, 6). Severe infection in SLE patients is also considered as a major cause of morbidity and mortality (41, 42). A retrospective analysis involving individuals with connective tissue disease (CTD) who were brought to the ICU due to sepsis from 2006 to 2019 showed that sepsis mortality was as high as 40.9% in patients with CTD (43). The important characteristics of rheumatic diseases are inflammation response and immune disorder (22, 44), which can be aggravated by infection, resulting in increased RDW and decreased albumin levels in the body. This could explain the conclusion of the present study in one way. The COX regression analyses performed in RA and SLE patients, respectively, yielded inconsistent conclusions. This inconsistency may be attributed to the limited sample size of SLE patients, which hindered the attainment of a statistically valid analysis. Furthermore, the differences in inflammatory processes between the two diseases may also contribute to the inconsistent findings. This needs to be verified by more large prospective studies and in-depth animal experiments.

The current study has several strengths. First, patients with rheumatism are easily overlooked in the ICU. In our research, critically ill patients with rheumatic diseases were taken as the research object to explore the correlation between RAR and all-cause mortality. Second, the sample size in our research is relatively large, and these data originate from MIMIC-IV database, a population-based, real-world study. Furthermore, RAR is a readily available and inexpensive parameter that can be applied in various clinical settings, especially in countries and regions where healthcare resources are scarce.

Despite this, certain restrictions must be taken into consideration. First, this study is a retrospective analysis, and selection bias and confounding bias will inevitably appear in this process. Furthermore, this study explored the correlation between RAR and clinical outcomes based on the initial value at ICU admission, and did not further evaluate the dynamic changes of RAR level. In addition, the MIMIC-IV database did not provide details on the specific causes of death for patients, thus our analysis was limited to all-cause mortality. Therefore, our findings are exploratory, and prospective studies with rigorous design in different settings are required to verify the conclusions of this study.

## Conclusion

In conclusion, the current evidence suggested that elevated RAR (> 6.07) was associated with all-cause mortality at 90-days and 360-days among critically ill patients with rheumatic diseases, serving as an independent risk factor for unfavorable prognosis.

## Data availability statement

The original contributions presented in the study are included in the article/Supplementary materials, further inquiries can be directed to the corresponding author.

## Ethics statement

The studies involving humans were approved by the Massachusetts Institute of Technology (Cambridge, MA) and Beth Israel Deaconess Medical Center (Boston, MA). The studies were conducted in accordance with the local legislation and institutional requirements. The ethics committee/institutional review board waived the requirement of written informed consent for participation from the participants or the participants’ legal guardians/next of kin because We had permission to access this database (certification number: 51774135; 36142713). All personal identifiers have already been deleted to afford privacy protection for participants. Consequently, this study waived consent requirements for individual patients.

## Author contributions

LY and QS contributed to the study conception and design. JM and LZ contributed to the data analysis. LY and JM wrote the first draft of the paper. QS critically reviewed, edited, and approved the manuscript. All authors contributed to the article and approved the submitted version.

## Supplementary material

Supplementary Figure 1 Comparison of AUCs for predicting 90-day all-cause mortality.

## References

[1] Keyßer G. Epidemiology and outcome of patients with rheumatic diseases in the intensive care unit. Z Rheumatol. (2019) 78:925–31. doi: 10.1007/s00393-019-00693-2, PMID: 31468166

[2] Peschken CA Hitchon CA Garland A Bernstein CN Chen H Fransoo R. A population-based study of intensive care unit admissions in rheumatoid arthritis. J Rheumatol. (2016) 43:26–33. doi: 10.3899/jrheum.150312, PMID: 26628597

[3] Moreels M Mélot C Leeman M. Prognosis of patients with systemic rheumatic diseases admitted to the intensive care unit. Intensive Care Med. (2005) 31:591–3. doi: 10.1007/s00134-005-2563-y, PMID: 15678307

[4] Larcher R Pineton de Chambrun M Garnier F Rubenstein E Carr J Charbit J. One-year outcome of critically ill patients with systemic rheumatic disease: a multicenter cohort study. Chest. (2020) 158:1017–26. doi: 10.1016/j.chest.2020.03.050, PMID: 32289313

[5] Arjmand M Shahriarirad R Shenavandeh S Fallahi MJ. Determination of the main causes, outcome, and prognostic factors of patients with rheumatologic diseases admitted to the medical intensive care unit in southern Iran. Clin Rheumatol. (2022) 41:3859–68. doi: 10.1007/s10067-022-06334-5, PMID: 35969279 PMC9376566

[6] Janssen NM Karnad DR Guntupalli KK. Rheumatologic diseases in the intensive care unit: epidemiology, clinical approach, management, and outcome. Crit Care Clin. (2002) 18:729–48. doi: 10.1016/S0749-0704(02)00025-8, PMID: 12418438

[7] Lippi G Plebani M. Red blood cell distribution width (RDW) and human pathology. One size fits all. Clin Chem Lab Med. (2014) 52:1247–9. doi: 10.1515/cclm-2014-0585, PMID: 24945432

[8] Bazick HS Chang D Mahadevappa K Gibbons FK Christopher KB. Red cell distribution width and all-cause mortality in critically ill patients. Crit Care Med. (2011) 39:1913–21. doi: 10.1097/CCM.0b013e31821b85c6, PMID: 21532476 PMC4427349

[9] Horta-Baas G Romero-Figueroa M. Clinical utility of red blood cell distribution width in inflammatory and non-inflammatory joint diseases. Int J Rheum Dis. (2019) 22:47–54. doi: 10.1111/1756-185X.13332, PMID: 30168259

[10] Shi S Chen L Gui X Chen L Qiu X Yu M. Association of red blood cell distribution width levels with connective tissue disease-associated interstitial lung disease (CTD-ILD). Dis Markers. (2021) 2021:1–7. doi: 10.1155/2021/5536360, PMID: 34457089 PMC8397563

[11] Targoński R Sadowski J Starek-Stelmaszczyk M Targoński R Rynkiewicz A. Prognostic significance of red cell distribution width and its relation to increased pulmonary pressure and inflammation in acute heart failure. Cardiol J. (2020) 27:394–403. doi: 10.5603/CJ.a2018.0103, PMID: 30234900 PMC8016006

[12] Eckart A Struja T Kutz A Baumgartner A Baumgartner T Zurfluh S. Relationship of nutritional status, inflammation, and serum albumin levels during acute illness: a prospective study. Am J Med. (2020) 133:713–722.e7. doi: 10.1016/j.amjmed.2019.10.031, PMID: 31751531

[13] Sheinenzon A Shehadeh M Michelis R Shaoul E Ronen O. Serum albumin levels and inflammation. Int J Biol Macromol. (2021) 184:857–62. doi: 10.1016/j.ijbiomac.2021.06.140, PMID: 34181998

[14] Arnau-Barrés I Güerri-Fernández R Luque S Sorli L Vázquez O Miralles R. Serum albumin is a strong predictor of sepsis outcome in elderly patients. Eur J Clin Microbiol Infect Dis. (2019) 38:743–6. doi: 10.1007/s10096-019-03478-2, PMID: 30680575

[15] Jin X Li J Sun L Zhang J Gao Y Li R. Prognostic value of serum albumin level in critically ill patients: observational data from large intensive care unit databases. Front Nutr. (2022) 9:770674. doi: 10.3389/fnut.2022.770674, PMID: 35769376 PMC9234460

[16] Xu W Huo J Chen G Yang K Huang Z Peng L. Association between red blood cell distribution width to albumin ratio and prognosis of patients with sepsis: a retrospective cohort study. Front Nutr. (2022) 9:1019502. doi: 10.3389/fnut.2022.1019502 36211519 PMC9539557

[17] Lu C Long J Liu H Xie X Xu D Fang X. Red blood cell distribution width-to-albumin ratio is associated with all-cause mortality in cancer patients. J Clin Lab Anal. (2022) 36:e24423. doi: 10.1002/jcla.24423 35396747 PMC9102686

[18] Seo YJ Yu J Park JY Lee N Lee J Park JH. Red cell distribution width/albumin ratio and 90-day mortality after burn surgery. Burns Trauma. (2022) 10:tkab050. doi: 10.1093/burnst/tkab050, PMID: 35097135 PMC8793164

[19] Hong J Hu X Liu W Qian X Jiang F Xu Z. Impact of red cell distribution width and red cell distribution width/albumin ratio on all-cause mortality in patients with type 2 diabetes and foot ulcers: a retrospective cohort study. Cardiovasc Diabetol. (2022) 21:91. doi: 10.1186/s12933-022-01534-4 35658957 PMC9166463

[20] Ni Q Wang X Wang J Chen P. The red blood cell distribution width-albumin ratio: a promising predictor of mortality in heart failure patients – a cohort study. Clin Chim Acta. (2022) 527:38–46. doi: 10.1016/j.cca.2021.12.027, PMID: 34979101

[21] Yoo JW Ju S Lee SJ Cho YJ Lee JD Kim HC. Red cell distribution width/albumin ratio is associated with 60-day mortality in patients with acute respiratory distress syndrome. Infect Dis (Lond). (2020) 52:266–70. doi: 10.1080/23744235.2020.1717599, PMID: 31996066

[22] Miglioranza Scavuzzi B Holoshitz J. Endoplasmic reticulum stress, oxidative stress, and rheumatic diseases. Antioxidants (Basel). (2022) 11:1306. doi: 10.3390/antiox11071306, PMID: 35883795 PMC9312221

[23] Johnson A Bulgarelli L Pollard T Horng S Celi LA Mark R. MIMIC-IV version 2.0. PhysioNet. (2022). doi: 10.13026/7vcr-e114

[24] von Elm E Altman DG Egger M Pocock SJ Gøtzsche PC Vandenbroucke JP. The strengthening the reporting of observational studies in epidemiology (STROBE) statement: guidelines for reporting observational studies. Int J Surg. (2014) 12:1495–9. doi: 10.1016/j.ijsu.2014.07.013, PMID: 25046131

[25] Jelkmann W. Proinflammatory cytokines lowering erythropoietin production. J Interf Cytokine Res. (1998) 18:555–9. doi: 10.1089/jir.1998.18.555, PMID: 9726435

[26] Weiss G Goodnough LT. Anemia of chronic disease. N Engl J Med. (2005) 352:1011–23. doi: 10.1056/NEJMra041809, PMID: 15758012

[27] Lippi G Targher G Montagnana M Salvagno GL Zoppini G Guidi GC. Relation between red blood cell distribution width and inflammatory biomarkers in a large cohort of unselected outpatients. Arch Pathol Lab Med. (2009) 133:628–32. doi: 10.5858/133.4.628, PMID: 19391664

[28] Salvagno GL Sanchis-Gomar F Picanza A Lippi G. Red blood cell distribution width: a simple parameter with multiple clinical applications. Crit Rev Clin Lab Sci. (2015) 52:86–105. doi: 10.3109/10408363.2014.992064 25535770

[29] Sudnitsyna J Skverchinskaya E Dobrylko I Nikitina E Gambaryan S Mindukshev I. Microvesicle formation induced by oxidative stress in human erythrocytes. Antioxidants (Basel). (2020) 9:929. doi: 10.3390/antiox9100929, PMID: 32998418 PMC7650597

[30] Brun JF Varlet-Marie E Myzia J Raynaud de Mauverger E Pretorius E. Metabolic influences modulating erythrocyte deformability and eryptosis. Metabolites. (2021) 12:4. doi: 10.3390/metabo12010004 35050126 PMC8778269

[31] Hu ZD Lippi G Montagnana M. Diagnostic and prognostic value of red blood cell distribution width in sepsis: a narrative review. Clin Biochem. (2020) 77:1–6. doi: 10.1016/j.clinbiochem.2020.01.001, PMID: 31935355

[32] He Y Liu C Zeng Z Ye W Lin J Ou Q. Red blood cell distribution width: a potential laboratory parameter for monitoring inflammation in rheumatoid arthritis. Clin Rheumatol. (2018) 37:161–7. doi: 10.1007/s10067-017-3871-7, PMID: 29101675

[33] Ustaoglu M Aktas G Avcioglu U Bas B Bahceci BK. Elevated platelet distribution width and red cell distribution width are associated with autoimmune liver diseases. Eur J Gastroenterol Hepatol. (2021) 33:e905–905e908. doi: 10.1097/MEG.0000000000002296, PMID: 34643621

[34] Hu ZD Chen Y Zhang L Sun Y Huang YL Wang QQ. Red blood cell distribution width is a potential index to assess the disease activity of systemic lupus erythematosus. Clin Chim Acta. (2013) 425:202–5. doi: 10.1016/j.cca.2013.08.007, PMID: 23954839

[35] Xu HJ Ma Y Deng F Ju WB Sun XY Wang H. The prognostic value of C-reactive protein/albumin ratio in human malignancies: an updated meta-analysis. Onco Targets Ther. (2017) 10:3059–70. doi: 10.2147/OTT.S137002 28790840 PMC5488759

[36] Gebreselassie A Aduli F Howell CD. Rheumatologic diseases and the liver. Clin Liver Dis. (2019) 23:247–61. doi: 10.1016/j.cld.2018.12.007, PMID: 30947875

[37] Yu H Han H Li J Li D Jiang L. Alpha-hydroxybutyrate dehydrogenase as a biomarker for predicting systemic lupus erythematosus with liver injury. Int Immunopharmacol. (2019) 77:105922. doi: 10.1016/j.intimp.2019.105922, PMID: 31669891

[38] Klinkmann G Waterstradt K Klammt S Schnurr K Schewe JC Wasserkort R. Exploring albumin functionality assays: a pilot study on sepsis evaluation in intensive care medicine. Int J Mol Sci. (2023) 24:12551. doi: 10.3390/ijms241612551, PMID: 37628734 PMC10454468

[39] Zhou D Wang J Li X. The red blood cell distribution width-albumin ratio was a potential prognostic biomarker for diabetic ketoacidosis. Int J Gen Med. (2021) 14:5375–80. doi: 10.2147/IJGM.S327733 34522133 PMC8434876

[40] Chabert P Danjou W Mezidi M Berthiller J Bestion A Fred AA. Short- and long-term prognosis of acute critically ill patients with systemic rheumatic diseases: a retrospective multicentre study. Medicine (Baltimore). (2021) 100:e26164. doi: 10.1097/MD.0000000000026164, PMID: 34477112 PMC8415942

[41] Tselios K Gladman DD Sheane BJ Su J Urowitz M. All-cause, cause-specific and age-specific standardised mortality ratios of patients with systemic lupus erythematosus in Ontario, Canada over 43 years (1971–2013). Ann Rheum Dis. (2019) 78:802–6. doi: 10.1136/annrheumdis-2018-214802, PMID: 30992296

[42] Barber M Clarke AE. Systemic lupus erythematosus and risk of infection. Expert Rev Clin Immunol. (2020) 16:527–38. doi: 10.1080/1744666X.2020.1763793, PMID: 32478627

[43] Krasselt M Baerwald C Petros S Seifert O. Sepsis mortality is high in patients with connective tissue diseases admitted to the intensive care unit (ICU). J Intensive Care Med. (2022) 37:401–7. doi: 10.1177/0885066621996257, PMID: 33631998 PMC8772250

[44] Aringer M. Inflammatory markers in systemic lupus erythematosus. J Autoimmun. (2020) 110:102374. doi: 10.1016/j.jaut.2019.102374, PMID: 31812331

