# Supplementary figures and images for: The correlation between red cell distribution width to albumin ratio and all-cause mortality in critically ill patients with rheumatic diseases: a population-based retrospective study
Source: Front Med (Lausanne). 2023 Oct 16;10:1199861. doi: 10.3389/fmed.2023.1199861 (PMC10614050; doi:10.3389/fmed.2023.1199861)

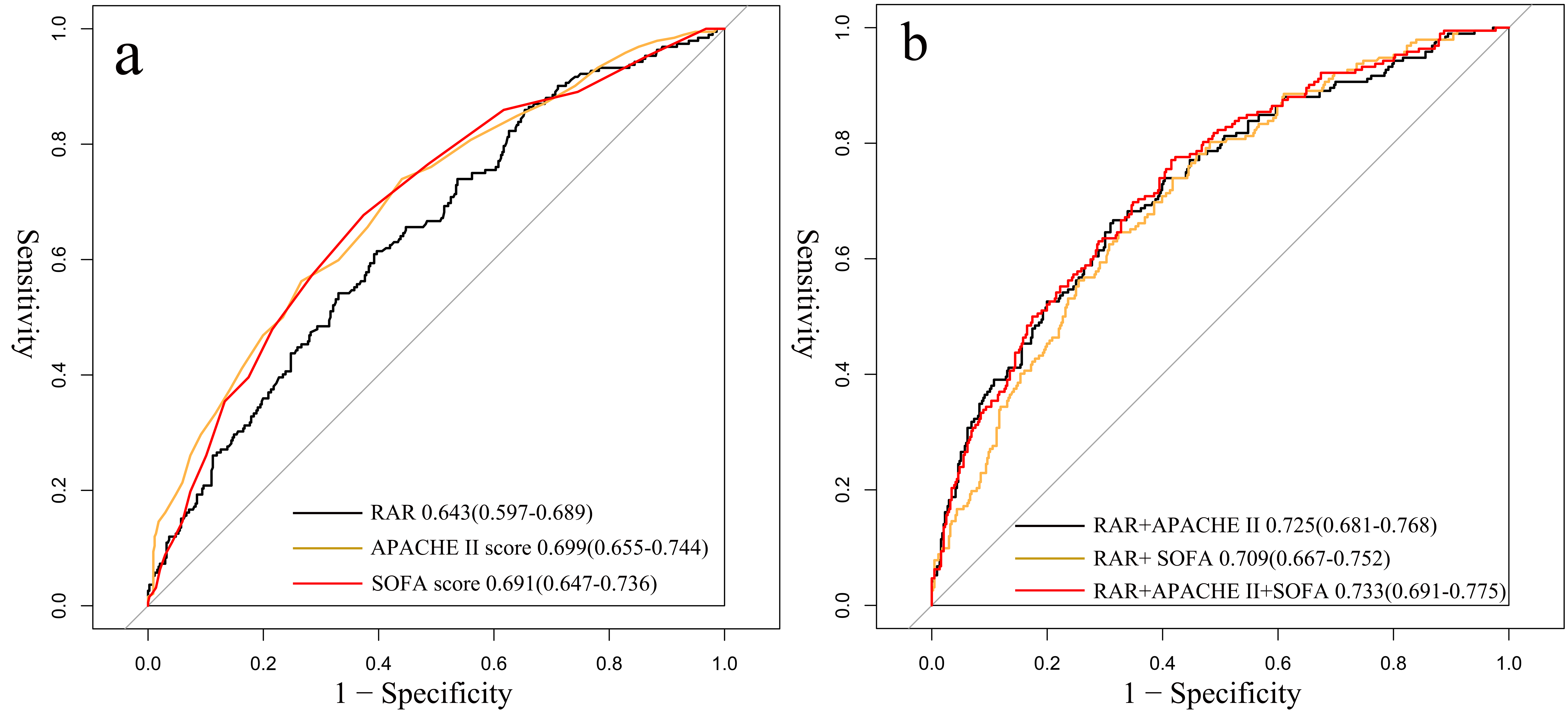

Supplement: Supplementary Figure 1 — Comparison of AUCs for predicting 90-day all-cause mortality. [file Image_1.TIF]
