# Supplementary material for: The correlation between red cell distribution width to albumin ratio and all-cause mortality in critically ill patients with rheumatic diseases: a population-based retrospective study
Source: Front Med (Lausanne). 2023 Oct 16;10:1199861. doi: 10.3389/fmed.2023.1199861 (PMC10614050; doi:10.3389/fmed.2023.1199861)
Supplement: Supplementary file 2 [file Data_Sheet_1.zip › Supplementary Table 4.DOCX]

**Supplementary Table 4** Sensitivity analysis after excluding patients with cirrhosis.

| **Variable** | **model I** | | |  | **model II** | | |  | **model III** | | |
| --- | --- | --- | --- | --- | --- | --- | --- | --- | --- | --- | --- |
|  | HR | 95%CI | *P* |  | HR | 95%CI | *P* |  | HR | 95%CI | *P* |
| **90-day mortality** |  |  |  |  |  |  |  |  |  |  |  |
| RAR <4.63 | baseline |  |  |  | baseline |  |  |  | baseline |  |  |
| RAR 4.63- 6.07 | 1.599 | 1.078-2.372 | 0.020 |  | 1.442 | 0.968-2.146 | 0.072 |  | 1.302 | 0.869-1.951 | 0.201 |
| RAR >6.07 | 2.377 | 1.631-3.464 | <0.001 |  | 1.752 | 1.193-2.574 | 0.004 |  | **1.921** | **1.288-2.865** | **<0.001** |
| **360-day mortality** |  |  |  |  |  |  |  |  |  |  |  |
| RAR <4.63 | baseline |  |  |  | baseline |  |  |  | baseline |  |  |
| RAR 4.63- 6.07 | 1.747 | 1.223-2.495 | 0.002 |  | 1.573 | 1.098-2.252 | 0.014 |  | 1.482 | 1.028-2.134 | 0.035 |
| RAR >6.07 | 2.564 | 1.819-3.615 | <0.001 |  | 1.951 | 1.374-2.770 | <0.001 |  | **2.201** | **1.530-3.167** | **<0.001** |

Model I was adjusted for no variables.

Model II was adjusted for malignancy, CKD, CHF, AKI, sepsis, MV use, and CRRT use.

Model III was adjusted for model II plus age, SOFA score, WBC, Scr, BUN, AG, potassium, phosphorus.

HR, hazard ratio; CI, confidence interval; RAR, red blood cell distribution width to albumin ratio; CKD, chronic kidney disease; CHF, congestive heart failure; AKI, acute kidney injury; MV, mechanical ventilation; CRRT, continuous renal replacement therapy; SOFA, sequential organ failure assessment; WBC, white blood cell; Scr, serum creatinine; BUN, blood urea nitrogen; AG, anion gap.
