# Supplementary material for: The correlation between red cell distribution width to albumin ratio and all-cause mortality in critically ill patients with rheumatic diseases: a population-based retrospective study
Source: Front Med (Lausanne). 2023 Oct 16;10:1199861. doi: 10.3389/fmed.2023.1199861 (PMC10614050; doi:10.3389/fmed.2023.1199861)
Supplement: Supplementary file 2 [file Data_Sheet_1.zip › Supplementary Table 5.DOCX]

**Supplementary Table 5** Comparison of the prognostic performance of RAR, APACHE II score, SOFA score and the combined indicators in patients.

| Variables | AUC | Cut-off | Sensitivity | Specificity | PPV | NPV | PLR | NLR |
| --- | --- | --- | --- | --- | --- | --- | --- | --- |
| RAR | 0.643 | 5.453 | 0.615 | 0.603 | 0.405 | 0.780 | 1.549 | 0.639 |
| APACHE II score | 0.699 | 21.500 | 0.678 | 0.560 | 0.425 | 0.830 | 1.679 | 0.465 |
| SOFA score | 0.691 | 5.500 | 0.677 | 0.626 | 0.444 | 0.815 | 1.811 | 0.516 |
| RAR+APACHE II score | 0.725 | 0.302 | 0.667 | 0.686 | 0.483 | 0.824 | 2.122 | 0.486 |
| RAR+SOFA score | 0.709 | 0.243 | 0.781 | 0.541 | 0.429 | 0.849 | 1.703 | 0.404 |
| RAR+APACHE II score+SOFA score | 0.733 | 0.255 | 0.771 | 0.585 | 0.450 | 0.853 | 1.857 | 0.392 |

RAR, red blood cell distribution width to albumin ratio; APACHE II, acute physiology and chronic health evaluation II; SOFA, sequential organ failure assessment; AUC, area under the curve; PPV, positive predictive value; NPV, negative predictive value; PLR, positive likelihood ratio; NLR, negative likelihood ratio.
