# Supplementary material for: The correlation between red cell distribution width to albumin ratio and all-cause mortality in critically ill patients with rheumatic diseases: a population-based retrospective study
Source: Front Med (Lausanne). 2023 Oct 16;10:1199861. doi: 10.3389/fmed.2023.1199861 (PMC10614050; doi:10.3389/fmed.2023.1199861)
Supplement: Supplementary file 2 [file Data_Sheet_1.zip › Supplementary Table 6.DOCX]

**Supplementary Table 6** Comparison of AUCs for predicting 90-day all-cause mortality.

| Variables | AUC (95% CI) | *P1* | *P2* |
| --- | --- | --- | --- |
| RAR | 0.643 (0.597-0.689) |  | <0.001 |
| APACHE II score | 0.699 (0.655-0.744) | 0.060 | 0.007 |
| SOFA score | 0.691 (0.647-0.736) | 0.103 | 0.015 |
| RAR+APACHE II score+SOFA score | 0.733 (0.691-0.775) | <0.001 |  |

AUC, area under the curve; CI, confidence interval; RAR, red blood cell distribution width to albumin ratio; APACHE II, acute physiology and chronic health evaluation II; SOFA, sequential organ failure assessment; P1, p-value for the equality compared to RAR; P2, p-value for the equality compared RAR+APACHE II score+SOFA score.
